# Efficacy of fixed-dose amlodipine and losartan combination compared with amlodipine monotherapy in stage 2 hypertension: a randomized, double blind, multicenter study

**DOI:** 10.1186/1756-0500-4-461

**Published:** 2011-10-28

**Authors:** Sung H Kim, Kyu H Ryu, Nam-Ho Lee, Jin-Ho Kang, Woo-Shik Kim, Sang-Weon Park, Hae-Young Lee, Jae-Joong Kim, Young-Keun Ahn, Soon Y Suh

**Affiliations:** 1 Department of Cardiology, Konkuk University School of Medicine, Seoul, Korea; 2 Cardiology Department, Hallym University Kangnam Sacred Heart Hospital, Gyeonggi, Korea; 3 Cardiology Department, Kangbuk Samsung Hospital, Seoul, Korea; 4 Cardiovascular Medicine Department, KyungHee University Medical Center, Seoul, Korea; 5 Cardiology Department, Korea University Anam Hospital, Seoul, Korea; 6 Cardiology Department, Seoul National University Hospital, Seoul, Korea; 7 Department of Cardiology, Asan Medical Center, Seoul, Korea; 8 Cardiology Department, Chonnam National University Hospital, Gwangju, Korea; 9 Gachon University Gil Medical Center, Incheon, Korea

**Keywords:** hypertension, amlodipine, losartan

## Abstract

**Background:**

The objective of this trial was to compare the blood-pressure lowering efficacy of amlodipine/losartan combination with amlodipine monotherapy after 6 weeks of treatment in Korean patients with stage 2 hypertension.

**Results:**

In this multi-center, double-blind, randomized study, adult patients (n = 148) with stage 2 hypertension were randomized to amlodipine 5 mg/losartan 50 mg or amlodipine 5 mg. After 2 weeks, patients with systolic blood pressure (SBP) > 140 mmHg were titrated to amlodipine 10 mg/losartan 50 mg or amlodipine 10 mg. After 4 weeks of titration, hydrochlorothiazide 12.5 mg could be optionally added to both groups. The change from baseline in SBP was assessed after 6 weeks. The responder rate (defined as achieving SBP < 140 mmHg or DBP < 90 mmHg) was also assessed at 2, 6 and 8 weeks as secondary endpoints. Safety and tolerability were assessed through adverse event monitoring and laboratory testing. Baseline demographics and clinical characteristics were generally similar between treatment groups. Least-square mean reduction in SBP at 6 weeks (primary endpoint) was significantly greater in the combination group (36.5 mmHg vs. 31.6 mmHg; p = 0.0117). The responder rate in SBP (secondary endpoints) was significantly higher in the combination group at 2 weeks (52.1% vs. 33.3%; p = 0.0213) but not at 6 weeks (p = 0.0550) or 8 weeks (p = 0.0592). There was no significant difference between groups in the incidence of adverse events.

**Conclusion:**

These results demonstrate that combination amlodipine/losartan therapy provides an effective and generally well-tolerated first line therapy for reducing blood pressure in stage 2 hypertensive patients.

**Trial Registration:**

ClinicalTrials.gov: NCT01127217

## Background

Hypertension has been recognized as an important risk factor for cardiovascular disease and is a leading risk factor for mortality [1]. Each year, the diagnosis and treatment of hypertension is increasing. By the year 2025, the prevalence is predicted to increase by 60% to approximately 1.56 billion worldwide, highlighting the need for improvement in the management and prevention of hypertension [2]. Inadequate recognition of hypertension, poor compliance of patients taking multiple drugs, and the reluctance of physicians to intensify antihypertensive therapy may account for the increasing burden of disease [3-5]. One way to overcome these barriers is through development of various fixed dose combination agents to treat hypertension.

Current US and European guidelines for the treatment of stage 2 hypertension recommend early initiation of combination treatment consisting of two anti-hypertensive drugs from different therapeutic classes since most hypertensive patients require two or more anti-hypertensive drugs to achieve their target blood pressure level [3,6]. Combination treatment for hypertension as initial therapy may simplify treatment and improve drug compliance by reducing the burden of taking multiple drugs [3,6]. In addition to increasing compliance, combination therapy may have other advantages over monotherapy, such as synergistic mechanisms of action for controlling hypertension and reduced side effects. For example, the capillary edema resulting from preferential arteriolar vasodilatation by dihydropyridine calcium channel blockers (CCB) can be ameliorated by angiotensin-receptor blockers (ARBs) or angiotensin converting enzyme (ACE) inhibitors; and the stimulation of the rennin-angiotensin system (RAS) induced by CCBs with potent vasodilatory and intrinsic natriuretic effects may be blocked by ARB and ACE inhibitors, increasing the blood pressure lowering effect [7]. The fixed dose combination of losartan and amlodipine is among the newer antihypertensive combinations that have been extensively studied and shown to be effective in the management of hypertension [8,9]. The objective of this trial was to compare the blood pressure lowering efficacy and tolerability profile of the combination of amlodipine/losartan with amlodipine monotherapy after 6 weeks of treatment in patients with stage 2 hypertension.

## Methods

### Study population

This was an 8-week, double blind, randomized study conducted at 8 hospitals in Korea. The study protocol was approved by the Korean FDA and the local ethical review boards of each hospital (Konkuk University Medical Center, Hallym University Kangnam Sacred Heart Hospital, Kangbuk Samsung Hospital, KyungHee University Medical Center, Korea University Anam Hospital, Seoul National University Hospital, Asan Medical Center, and Chonnam National University Hospital). The study was conducted in accordance with the ethical principles of the current Declaration of Helsinki. Subjects signed informed consent prior to any relevant laboratory tests.

Adults aged 18 or older with stage 2 hypertension [diagnosed according to the criteria set forth in the 7^th ^Report of the Joint National Committee on Prevention, Detection, evaluation, and Treatment of High Blood Pressure (JNC 7)[3] were eligible for the study. Subjects on anti-hypertensive drugs were eligible if their sitting systolic blood pressure (SBP) was ≤180 mmHg and sitting diastolic blood pressure (DBP) was ≤110 mmHg. These subjects underwent a 3- to 7-day washout period prior to randomization. Subjects were randomized if they had SBP ≥ 160 mmHg and ≤199 mmHg and DBP ≥ 80 mmHg and ≤119 mmHg at randomization.

Subjects were excluded if they had variability of ≥ 20 mmHg in SBP or ≥ 10 mmHg in DBP in three measurements at screening. Subjects who had been treated with systemic steroid hormones, anesthetics, tri- & tetra-cyclic antidepressants, nonsteroid anti-inflammatory drugs, and/or oral contraceptives for 3 months, or had renal or hepatic disease, and women that were pregnant or nursing were also excluded from the study.

Subjects were randomized into two treatment groups: amlodipine 5 mg/losartan 50 mg or amlodipine 5 mg using an allocation ratio of 1:1. Subjects were instructed to take their corresponding investigational products with matching placebos for two weeks starting at randomization. Information on blinding was provided to the principal investigators in sealed forms. Sealed status was maintained until Korean FDA inspection. Unblinding was only considered in the event of a significant medical emergency.

All subjects had their blood pressure measured at each visit using mercury sphygmomanometers provided by the sponsor and the same investigator measured blood pressure at each visit, if possible. At screening, blood pressure was measured 3 times in both arms and the arm with higher mean SBP was selected as the reference arm. At the remaining visits, blood pressure was measured from the reference arm 3 times and the mean value was used. Caffeine, exercise and smoking were not allowed at least 30 minutes prior to blood pressure measurements.

At Week 2, subjects taking amlodipine 5 mg/losartan 50 mg with measured SBP ≥ 140 mmHg were prescribed the increased dose of amlodipine 10 mg/losartan 50 mg, while subjects taking amlodipine 5 mg with measured SBP ≥ 140 mmHg were prescribed the increased dose of amlodipine 10 mg for six weeks. Subjects with measured SBP < 140 mmHg maintained their existing dose of amlodipine for the remaining six weeks. At six weeks, the investigators could add hydrochlorothiazide (HCTZ) 12.5 mg to the treatment regimen if the measured SBP was ≥ 140 mmHg.

Compliance was checked and recorded at each visit following randomization. Actual doses were recorded by counting the number of tablets. If compliance was < 80% the subject was excluded from the Per Protocol (PP) population.

### Efficacy Assessment

The primary endpoint was the change from baseline in SBP after 6 weeks of treatment. Secondary endpoints were the change from baseline in SBP after 2 and 8 weeks of treatment, the change from baseline in DBP after 2, 6, and 8 weeks of treatment, and the responder rates, defined as the percentage of patients who achieved target blood pressure (SBP < 140 mmHg or DBP < 90 mmHg) or achieved a change from baseline in SBP or DBP that exceeded 20 mmHg and 10 mmHg, respectively.

### Safety Assessment

A safety evaluation was performed on all patients who were randomized and took at least one dose of study drug. Adverse events were assessed; and laboratory tests, including hematology, blood chemistry, and urine analysis, as well as physical examination, pulse and electrocardiogram were conducted.

### Statistical analysis

A sample size of 136 subjects was required for at least 85% power and a significance level of 0.05 to detect the difference in change from baseline between treatment groups. A standard deviation of 13.6 mmHg and a between group difference of 7.0 mmHg was assumed based on a previous phase II study (unpublished data). It was estimated that approximately 160 subjects (80 subjects in each treatment group) should be screened in order to reach 72 randomized subjects in each treatment group.

The primary population for analysis was the intent-to-treat (ITT) population, defined as all randomized patients who had a baseline blood pressure measurement and at least 1 post baseline efficacy measurement. Efficacy data were also analyzed using the PP population to ensure consistency. For continuous demographic variables, mean, SD, minimum and maximum values were determined and compared by T-test or Wilcoxon rank sum test. For categorical demographic variables, absolute and relative frequencies were determined and compared by χ^2^-test or Fisher's exact test. The primary efficacy variable (change in SBP from baseline to week 6) was analyzed using analysis of covariance (ANCOVA). The absolute and relative frequencies of SBP < 140 mmHg, DBP < 90 mmHg, SBP reduction > 20 mmHg, DBP reduction > 10 mmHg, and responder rate in terms of changes after 4 and 6 weeks of treatment relative to baseline were determined and compared by using χ^2^-test or Fisher's exact test. No adjustments were made for multiplicity.

## Results

### Patient Disposition and Baseline Characteristics

**Figure 1:**
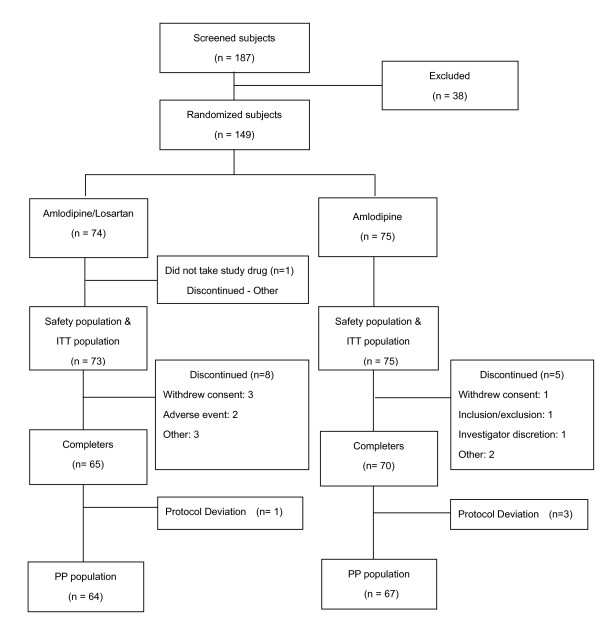
**Patients Disposition (ITT: intention to treatment, PP: Per protocol)**.

The flow of subjects through the study is shown in Figure 1. A total of 187 subjects were screened and 149 were randomized. One subject in the amlodipine/losartan combination group did not take study drug; therefore, 73 subjects were included in the amlodipine/losartan combination group and 75 were included in the amlodipine monotherapy group for the safety analysis and the ITT population. The PP population consisted of 131 subjects: 64 in the amlodipine/losartan combination group and 67 in the amlodipine monotherapy group (Figure 1). Fourteen subjects discontinued prior to completing the study. Sixty five subjects (87.8%) in the amlodipine/losartan group and 70 subjects (93.3%) in the amlodipine group completed the trial (p = 0.2503).

Demographics and baseline characteristics were similar between treatment groups except for the rates of concurrent alcohol drinking, which were significantly different between treatment groups (67.1%, 49/73 in the amlodipine/losartan combination group, and 84.0%, 63/75 in the amlodipine monotherapy group; p = 0.0499; Table 1). The mean SBP/DBP was 169.0/103.4 mmHg in the amlodipine/losartan combination group and 170.5/102.3 mmHg in the amlodipine monotherapy group.

**Table 1:** Demographics and Baseline Characteristics in the ITT population (ITT = 148 subjects)

	Amlodipine/Losartan	Amlodipine	p-value
		
	(N = 73)	(N = 75)	
Age (year)			0.6654^(a)^
Mean	54.9	55.6	
SD	11.0	9.7	
range(min~max)	46.0 (31.0~ 77.0)	45.0 (33.0~ 78.0)	

Gender			0.2964^(b)^
Male	52 (71.2)	59 (78.7)	
Female	21 (28.8)	16 (21.3)	

Height (cm)			0.7504^(a)^
Mean	166.2	165.8	
SD	7.9	6.9	

Body Weight (kg)			0.7702^(a)^
Mean	71.2	70.7	
SD	12.4	10.0	

Alcohol			0.0499^(b)^*
non alcohol drinker	19 (26.0)	10 (13.3)	
Historical alcohol drinker	5 (6.9)	2 (2.7)	
Alcohol drinker	49 (67.1)	63 (84.0)	

Smoking			0.3436^(b)^
Non smoker	37 (50.7)	32 (42.7)	
Historical smoker	14 (19.2)	22 (29.3)	
Smoker	22 (30.1)	21 (28.0)	

SBP (mmHg)			0.3466^(a)^
Mean	169.0	170.5	
SD	9.2	9.8	

DBP (mmHg)			0.4099^(a)^
Mean	103.4	102.3	
SD	8.68	8.0	

(a) Unpaired T-test

(b) Pearson's chi-square test

*Statistically significant difference

### Efficacy

**Figure 2:**
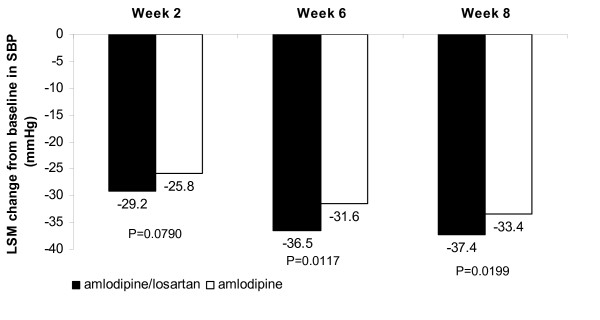
**Change from baseline in SBP (mmHg) in the ITT population (N = 148)**.

**Figure 3:**
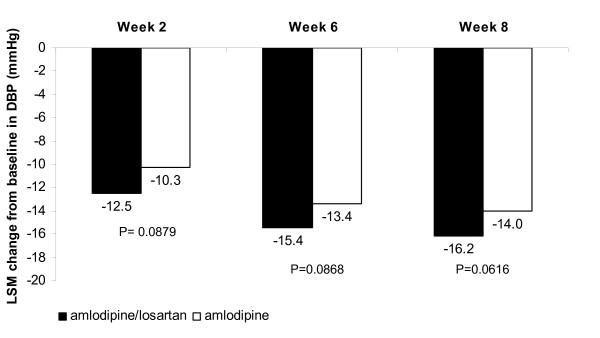
**Changes from baseline in DBP (mmHg) in the ITT population (N = 148)**.

The least-square mean change from baseline in SBP after 6 weeks was significantly greater in the amlodipine/losartan combination group (36.5 mmHg) compared with the amlodipine monotherapy group [(31.6 mmHg) difference = -4.94 mmHg (95% confidence interval: -8.76, -1.11 p = 0.0117)] (Figure 2). The results of the PP population analysis were consistent with the analysis of the ITT population. There was a significantly greater reduction from baseline in SBP in the amlodipine/losartan combination group compared with the amlodipine monotherapy group at Week 8 (p = 0.0199), and although the reduction was numerically greater in the amlodipine/losartan combination group compared with the amlodipine monotherapy group at week 2, the difference was not statistically significant (p = 0.0790; Figure 2). Although the difference in DBP reduction between the two groups increased continuously by treatment period until Week 8, there was no significant difference between groups at any time point (Figure 3).

**Figure 4:**
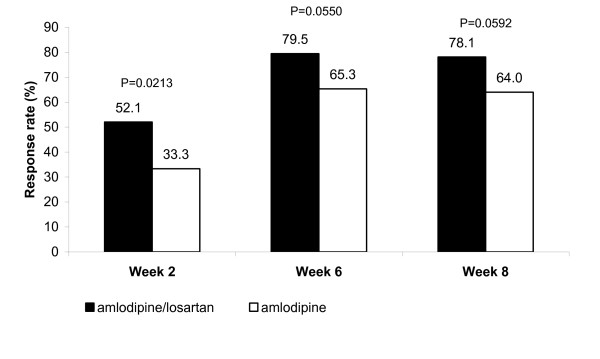
**Responder rate for SBP < 140 mmHg at Week 2, Week 6 and Week 8 (ITT population; N = 148)**.

At Week 2, 38/73 subjects (52.1%) in the amlodipine/losartan combination group compared with 25/75 subjects (33.3%) in the amlodipine monotherapy group achieved the target SBP < 140 mmHg, and this difference was significantly different (p = 0.0213; Figure 4). At Weeks 6 and 8, 58/73 subjects (79.5%) and 57/73 subjects (78.1%), respectively, in the amlodipine/losartan combination group compared with 49/75 subjects (65.3%) and 48/75 subjects (64.0%), respectively, in the amlodipine monotherapy group achieved the target SBP < 140 mmHg, and these differences were not significantly different (p = 0.0550 and p = 0.0592; Figure 4).

**Figure 5:**
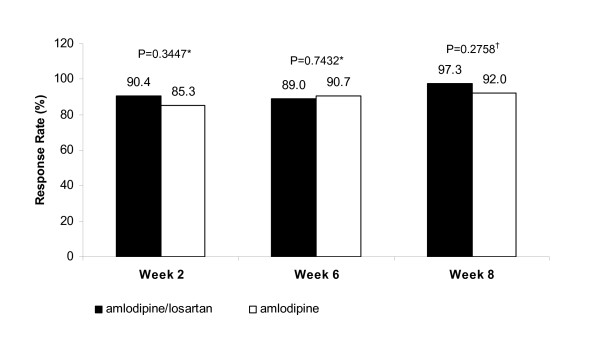
**Cumulative responder rate for subjects achieving SBP < 140 mmHg, DBP < 90 mmHg, change in SBP > 20 mmHg or change in DBP > 10 mmHg at Week 2, Week 6 and Week 8 (ITT population; N = 148)**.

Changes were similar between the two groups in the cumulative responder rate for target DBP level < 90 mmHg, SBP reduction > 20 mmHg, and DBP reduction > 10 mmHg at Week 2 (90.4% in the amlodipine/losartan combination group vs 85.3% in the amlodipine monotherapy group, p = 0.3447), at Week 6 (89.0% in the amlodipine/losartan combination group vs 90.7% in the amlodipine monotherapy group, p = 0.7432,) and at Week 8 (97.3% in the amlodipine/losartan combination group vs 92.0% in the amlodipine monotherapy group, p = 0.2758; Figure 5). At Weeks 2 and 6, the analysis results for the PP population were similar to those of the ITT population. However, at Week 8 in the PP population, the responder rate was significantly higher in the amlodipine/losartan combination group vs. the amlodipine monotherapy group (100.0% vs. 91.0%; p = 0.0280).

In ITT analysis group, the rate of subjects with dose escalation after 2 weeks of treatment in the amlodipine/losartan combination group (47.2%, 34/72) was lower than that of the amlodipine monotherapy group (64.8%, 46/71). The rate of subjects with treatment of HCTZ 12.5 mg after 6 weeks of treatment in the amlodipine/losartan combination group (16.7%, 11/66) was also lower than that of the amlodipine monotherapy group (31.4%, 22/70).

### Safety

A total of 86 treatment emergent adverse events (TEAE) were reported in 47 subjects [44 in 23 subjects (31.5%) in the amlodipine/losartan combination group and 42 in 24 subjects (32.0%) in the amlodipine monotherapy group; Table 2]. There was no significant difference in the incidence of reported adverse events between treatment groups. Most TEAEs were reported as mild. There were 3 subjects (4.1%) in the amlodipine/losartan group and 2 subjects (2.7%) in the amlodipine group that reported TEAEs considered moderate in intensity. There was one TEAE (1.3%) reported in the amlodipine monotherapy group that was considered severe. Thirty four adverse events considered related to study drug were reported: 14 in 8 subjects (11.0%) from the amlodipine/losartan group and 20 in 13 subjects (17.3%) from the amlodipine monotherapy group. The most frequently reported treatment related adverse events included dizziness, headache, somnolence, hot flush, and peripheral edema. Two subjects (2.7%) in the amlodipine/losartan combination group and 1 subject (1.3%) in the amlodipine monotherapy group reported serious adverse events. No deaths were reported during this trial (Table 2).

**Table 2:** Summary of Adverse Events

Adverse Events		Amlodipine/Losartan (N = 73)		Amlodipine (N = 75)		p-value
		
		n (%)	[number of reported AEs]	n (%)	[number of reported AEs]	
Treatment-Emergent Adverse Events		23 (31.5)	[44]	24 (32.0)	[42]	0.9486^(a)^
	Mild	20 (27.4)	[41]	21 (28.0)	[39]	1.0000^(b)^
Severity	Moderate	3 (4.1)	[3]	2 (2.7)	[2]	ns
	Severe	0	[0]	1 (1.3)	[1]	
Drug-related AE		8 (11.0)	[14]	13 (17.3)	[20]	0.2665^(a)^
Serious AE		2 (2.7)	[2]	1 (1.3)	[1]	0.6173^(b)^
AE which causes rule-out		2 (2.7)	[2]	0	[0]	0.2416^(b)^
Death		0	[0]	0	[0]	

(a) Pearson's chi-square test

(b) Fisher's exact test

## Discussion

The results of this study demonstrated that the amlodipine/losartan 5/50 mg combination was significantly more effective at reducing SBP than amlodipine monotherapy after 6 weeks of therapy in stage 2 hypertensive patients. Responder rates for SBP < 140 mmHg were significantly greater at Week 2 in the combination group, suggesting a more rapid onset of action, and were numerically greater in the combination group at Weeks 6 and 8, with p-values nearing statistical significance. Both treatments were generally well tolerated. These results support the recommendations of the JNC-7, the European Society of Hypertension, and the Taiwan Society of Cardiology Guidelines for the Management of Hypertension, which advocate early initiation of combination treatment consisting of two anti-hypertensive drugs with complimentary mechanisms of action for stage 2 hypertension patients [3,6,10]

Losartan and amlodipine are frequently used as first-line therapy in hypertensive patients, [9,11,12] and combining these two drugs has also been shown to be effective in lowering blood pressure [13,14]. The results of this study, in which a fixed dose combination was utilized, support those earlier studies and extend their findings to demonstrate significant reductions in blood pressure in patients with stage 2 hypertension. Of note, the rate of dose escalation, as well as the addition of HCTZ, was lower in the amlodipine/losartan combination group compared with the amlodipine monotherapy group after 2 weeks of treatment. This is consistent with the results of the responder rates which showed greater achievement of SBP < 140 mmHg with the combination treatment vs. monotherapy at Week 2 and supports the use of the combination regimen as a first-line therapy in patients with stage 2 hypertension. Not only was the improvement in SBP greater with the combination, the number of drugs required to achieve target was limited to 1 (rather than 2 or more) for the majority of patients on combination throughout the trial. Therefore, these results provide support for the fixed dose combination of amlodipine/losartan 10/50 as an effective and generally well-tolerated first-line option for patients with stage 2 hypertension through the potential improvement of compliance and rapid onset of action.

Several different combination therapies have been shown to effectively treat hypertension. In two separate studies, the combination CCB-ACE inhibitor benazepril/amlodipine was shown to be significantly more effective in reducing blood pressure, and more patients achieved blood pressure control compared with amlodipine monotherapy [15,16]. Similar results were observed in a study of the CCB-ACE inhibitor combination enalapril/felodipine [17]. In the ACCOMPLISH trial, it was reported that the CCB-RAS inhibitor combination benazepril/amlodipine was superior to the CCB-diuretic combination benazepril-hydrochlorothiazide in reducing cardiovascular events in patients with hypertension who were at high risk for such events [18]. However, ACE inhibitors affect kinin metabolism and are associated with a high incidence of dry cough. In order to avoid this side affect, ARBs have been combined with amlodipine to reduce blood pressure.

Several studies combining amlodipine with an ARB have demonstrated superior efficacy over monotherapy and comparable tolerability in patients of different races/ethnic origin. The EX-STAND trial showed that the combination therapy of amlodipine and valsartan (5/160 mg to 10/320 mg) achieved greater reductions in blood pressure after 8 weeks of treatment and faster onset of action in black subjects with stage 2 hypertension compared with corresponding component amlodipine monotherapies (33.3 mmHg vs. 26.6 mmHg, p < 0.001) [19]. In Indian subjects with stage 2 hypertension, the combination of telmisartan/amlodipine 40/5 mg was shown to be significantly more effective after 12 weeks of treatment (p < 0.05), and more patients achieved blood pressure control compared with amlodipine monotherapy (p < 0.05) [20]. The COACH study showed that combination olmesartan and amlodipine therapy (10, 20, 40/5, 10 mg) was significantly more effective than amlodipine monotherapy in mild to moderate hypertensive patients, of which 79.3% had stage 2 hypertension [21]

Although reductions in blood pressure may be affected by ethnic/racial difference and baseline blood pressure levels, the data from this study and the results from previous clinical trials demonstrate the efficacy of ARB and amlodipine combination therapy in treating stage 2 hypertension and support the recommendation that in patients who do not achieve recommended blood pressure reductions with a low dose of an antihypertensive agent, a combination therapy may be more effective than increasing the dose of a single agent. However, a more cost-effective option may be to use combination therapy first-line to achieve blood pressure goals quickly. The ability to generalize these results to the population as a whole, and especially to older patients, is limited by the small number of patients included and by the lack of ethnic diversity. Larger studies are warranted in more diverse populations. The results of the secondary and exploratory endpoints should be interpreted with caution since no adjustments were made for multiplicity.

## Conclusions

In conclusion, the results of this trial support the use of early initiation of amlodipine/losartan combination followed by subsequent dose escalation in patients who have not achieved recommended blood pressure levels. Amlodipine/losartan was generally well-tolerated and provides a superior first-line therapeutic option for patients with stage 2 hypertension.

## Competing interests

The authors declare that they have no competing interests.

## Authors' contributions

All authors participated in acquisition and interpretation of data, critically reviewing and revising the manuscript for important intellectual content, and approved the final version for publication.
